# Case report: Therapeutic potential of T-VEC in combination with MEK inhibitors in melanoma patients with NRAS mutation

**DOI:** 10.3389/fonc.2023.1111119

**Published:** 2023-03-07

**Authors:** Sonja C. S. Simon, Verena Müller, Jochen S. Utikal

**Affiliations:** ^1^ Skin Cancer Unit, German Cancer Research Center (DKFZ), Heidelberg, Germany; ^2^ Department of Dermatology, Venereology and Allergology, University Medical Center Mannheim, University of Heidelberg, Mannheim, Germany; ^3^ German Cancer Research Center (DKFZ) Hector Cancer Institute, University Medical Center Mannheim, Mannheim, Germany

**Keywords:** melanoma, T-VEC, MEK inhibitor, NRAS mutation, case series

## Abstract

Mutations in the *NRAS* gene are common alterations in malignant melanoma. However, there are no specific treatment options approved for *NRAS*-mutated melanoma patients besides immune checkpoint inhibition. Since preclinical data suggests a synergistic effect of a MEK inhibitor (MEKi) and the oncolytic virus talimogene laherparepvec (T-VEC), we have treated three melanoma patients with this combination. All of the three patients had been suffering from recurring cutaneous and subcutaneous in-transit metastases. Upon treatment one patient (case 1) presented full regression of locoregional metastases and remained progression-free until date, for almost three years. The second patient (case 2) showed a partial regression of painful gluteal satellite metastases but died from brain metastases. The third patient (case 3) showed a durable response of locoregional metastases for seven months. The combination treatment was well tolerated with common adverse events known for each single agent. This report is the first case series presenting a clinical benefit of the combined T-VEC and MEKi treatment. We suggest the combination of T-VEC and MEKi as an off-label treatment option for patients with *NRAS* mutations, especially with recurrent in-transit or satellite metastases.

## 1 Introduction

Malignant melanoma is one of the most prevalent and most aggressive skin cancers. Genetic alterations are common in melanoma with *BRAF* being the most frequently mutated gene. Moreover, about 20% of melanomas have mutations in the *NRAS* gene. *NRAS* mutated melanomas are often more aggressive and associated with a higher risk for disease progression compared to non-*NRAS*-mutated melanomas (1). Besides treatment with an immune checkpoint inhibitor (ICI), the combination of a BRAF inhibitor and a MEK inhibitor (MEKi) is the approved therapeutic option for *BRAF*-mutated melanomas. Although the therapeutic opportunities have been revolutionized during the last decade and *NRAS* mutations are frequent in melanoma, there are still no further approved treatment options for *NRAS*-mutated melanomas (2).

In daily clinical routine, the shortage of therapeutic agents and the urgent need for more treatment options for patients with an *NRAS* mutation is often apparent after tumor progression under an ICI.

Another approach to treat melanoma patients with non-resectable metastases without bone, brain, lung or visceral involvement is the oncolytic virus T-VEC. This agent is applied intralesional and is often used for cutaneous as well as subcutaneous metastases. Preclinical data suggests a synergistic effect on the anti-tumoral response of talimogene laherparepvec T-VEC together with a MEKi (3). Therefore, we treated three melanoma patients harboring a *NRAS* mutation and uncontrolled in-transit or satellite metastatic spread with the combination of T-VEC and a MEKi (cobimetinib or trametinib).

## 2 Case descriptions

### 2.1 Case 1

**Figure 1:**
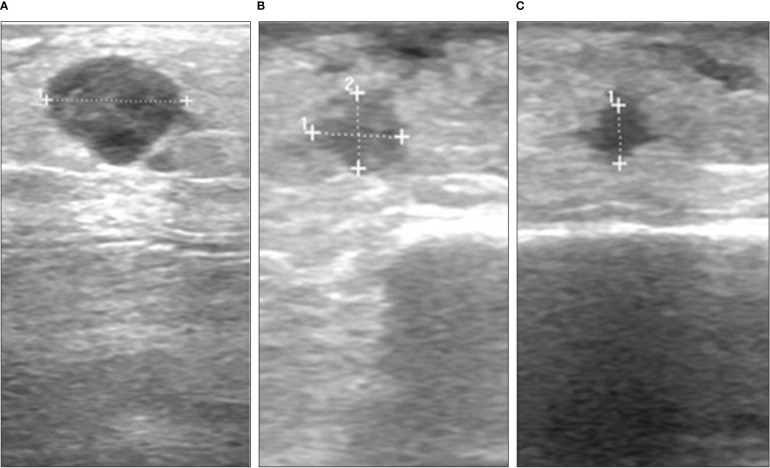
Course of a subcutaneous melanoma metastasis under combination of MEK inhibitor and T-VEC in case 1. Ultrasound images from **(A)** April 2017, **(B)** April 2018 and **(C)** January 2019.

A 74-year old female was diagnosed with malignant melanoma in 2011. The primary tumor on the left lower leg had a tumor depth of 4.9 mm without ulceration (pT4a). A mutation analysis showed a *NRAS* Q61L mutation and a *BRAF* wildtype. After a positive sentinel lymph node biopsy of the left groin, a lymph node dissection was performed and revealed no further nodal involvement of the melanoma (pN1a). The staging by MRI of the whole body did not show any further metastasis (cM0). The patient was included in the EORTC-18071 trial and received an adjuvant treatment with four cycles of ipilimumab until September 2011. Between 2012 and 2015, the patient presented a total of 13 cutaneous and subcutaneous, satellite and in-transit metastases on the left lower leg. Each metastasis was resected in toto. After five new in-transit metastases in July 2015, we decided to start a treatment with nivolumab (qW4). After two cycles of nivolumab, the patient developed a rash and arthralgia which led to discontinuation of the treatment. In December 2015, the patient presented with two in-transit metastases once more, which were not suitable for radiation or isolated limb perfusion due to wound infections. Therefore, we decided to reinitiate ICI treatment with pembrolizumab (qW3) in January 2016. The treatment was stopped after 4 cycles of pembrolizumab because of diarrhoea and arthralgia. In April 2016, the patient progressed with iliac lymph node metastases on the left side and a radiation therapy (60Gy) was performed. After 8 months, the patient presented again with five new subcutaneous in-transit metastases of the left leg and left parailiac lymph node metastases in the whole-body MRI. Facing this progressive disease, a systemic treatment with ICI was inapplicable because of adverse events and a conventional targeted therapy was ineligible because the tumor was *BRAF* wildtype. Due to the known *NRAS* Q61L mutation, we considered an off-label treatment with a MEKi. A treatment with the MEKi cobimetinib was started with a dose of 60mg per day from day 1 until day 21 followed by a seven-day break in a 28-day cycle. After three cycles of cobimetinib, the patient developed a rash resistant to local steroids; the dose was therefore decreased to 40mg per day. The following staging showed a regression of the left parailiac lymph node metastases, but also revealed two more new in-transit metastases. Therefore, we decided to try another local approach and in April 2017 we started to combine the MEKi treatment with the application of T-VEC (**Figure 1A**, **Supplementary Table 1**). Over the course of two years, the in-transit metastases regressed constantly (**Figures 1B, C**) and no further in-transit metastases developed under the combination therapy. In July 2019, the treatment with T-VEC was stopped since no local or distant metastases were detectable in a PET-CT. The treatment with cobimetinib was continued for two more months and stopped due to recurrent falls. In the follow-up imaging until today, for almost three years, the patient has stayed free from any metastases.

### 2.2 Case 2

**Figure 2:**
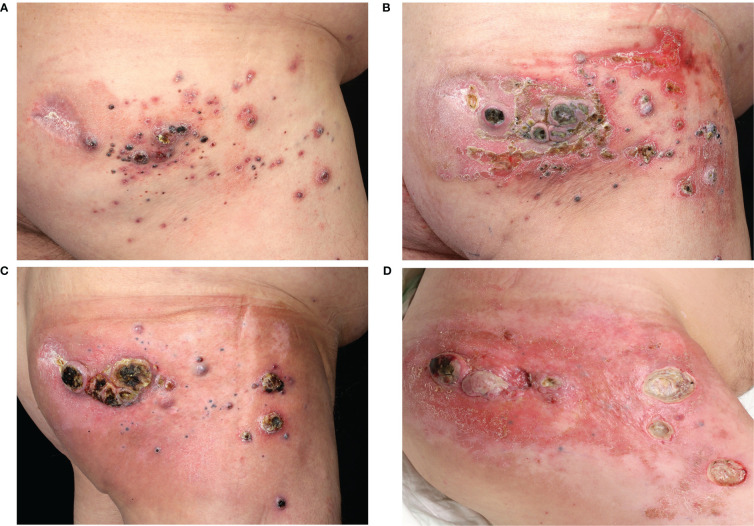
Course of cutaneous melanoma metastases in case 2. Clinical images from **(A)** November 2018, **(B)** January 2019, **(C)** March 2019 and **(D)** August 2019.

A 55-year-old male patient was diagnosed with malignant melanoma of the right gluteal region in April 2018. The primary tumor had a depth of 7.0 mm without ulceration (pT4a). The initial staging by CT of the whole body showed lymph node metastases in the right inguinal region (cN2b). A lymph node dissection and adjuvant radiotherapy were planned. However, three days before the dissection appointment, the patient presented with cutaneous satellite metastases on the right gluteal region. Because of the widespread and rapid growth of satellite metastases, the patient was included in the Imspire trial and received four cycles of pembrolizumab (qW3). In September 2018, the patient presented progressive cutaneous satellite and in-transit metastases and therefore received laser treatment and excisions of multiple metastases as well as postoperative local treatment with imiquimod. The mutation analysis of a metastasis showed a *NRAS* Q61K mutation and a *BRAF* wildtype. The CT scan revealed progressive lymph node metastases in the right inguinal and mesenterial region. A radiotherapy of the lymph node metastases (3x8 Gy) and cutaneous metastases (3x8 Gy and 45x3 Gy) combined with hyperthermia treatment was performed. Furthermore, the patient started a treatment with ipilimumab in December 2018. After one administration of ipilimumab, the patient presented with reduced condition and hyperglycemia. An immune-related hypophysitis and diabetes was diagnosed and the ipilimumab-treatment was stopped. Facing rapidly progressive cutaneous satellite metastases (**Figures 2A, B**), we initiated a local treatment with one T-VEC injection. No further injections were conducted for eight weeks since the patient was screened for a clinical trial. During this period, the satellite metastases showed no sign of regression. The staging in February 2019 revealed a progressive disease with a new cerebral metastasis. The patient received gamma knife radiosurgery. In addition, we considered an off-label treatment with a MEKi because of the known *NRAS* Q61K mutation. In March 2019, the treatment was started with trametinib 2mg per day. The treatment with T-VEC was continued in combination with trametinib (**Supplementary Table 1**). Adverse events under the combination treatment were fever responsive to paracetamol after the T-VEC injections and a rash responsive to local steroids and doxycycline. In the middle of March 2019, after three injections of T-VEC and six weeks of trametinib, a regression of the cutaneous metastases was visible (**Figure 2C**). Due to a recurrent rash and progressive swellings of the right leg, trametinib was paused and re-induced with a dose of 1mg per day and later on with 0,5mg per day. The staging in June 2019 showed progressive cerebral and lymph node metastases in the right inguinal and parailiac region. Radiotherapy of the lymph node and cutaneous metastases as well as gamma knife radiosurgery of the cerebral metastases was performed. A treatment with nivolumab (qw4) was started while the treatment with T-VEC was continued in combination. In August 2019, three symptomatic gluteal metastases were resected (**Figure 2D**) and the patient presented with an erysipelas of the gluteal region. The local treatment with T-VEC was stopped. The following staging in September 2019 revealed a progressive disease with stable cerebral metastases but progressive intramuscular and intestinal metastases. Best supportive care was discussed with the patient, who decided for another tumor debulking on the right gluteal and upper leg region. A CT scan in January 2020 revealed another massive progression of all metastases and the patient died in February 2020.

### 2.3 Case 3

**Figure 3:**
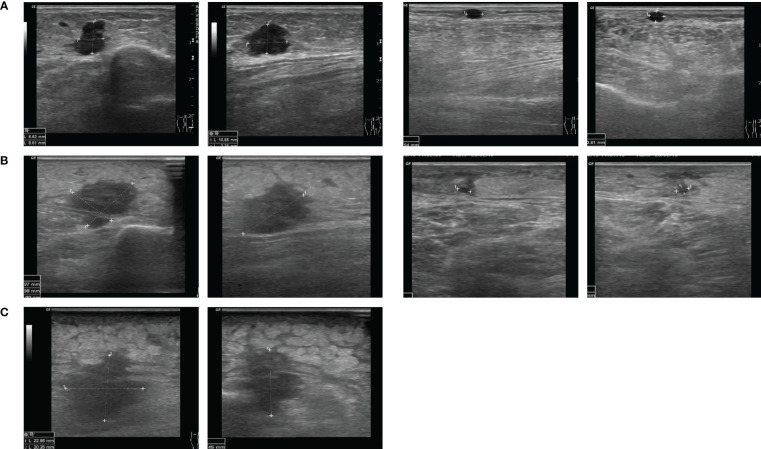
Course of subcutaneous melanoma metastases under combination of MEK inhibitor and T-VEC in case 3. Ultrasound images from **(A)** August 2018, **(B)** February 2019 and **(C)** July 2019.

A 79-year-old female patient was diagnosed with malignant melanoma on the left lower limb in July 2009. The primary tumor had a depth of 2.0 mm without ulceration (pT2a). After a positive sentinel lymph node biopsy of the left groin, she received a lymph node dissection which showed no further nodal involvement of the melanoma (pN1a). The following adjuvant treatment with interferon-α was stopped due to adverse events. From 2009 to 2015, the patient developed seven cutaneous and subcutaneous satellite and in-transit metastases on the left lower limb. All of them were R0-resected. In April 2007, the ultrasound of the right groin showed a lymph node metastasis, which was extirpated. A mutation analysis of another cutaneous in-transit metastasis showed a *NRAS* Q61L mutation and a *BRAF* wildtype. Until July 2018, the patient presented with four more in-transit metastases on the left lower limb which were excised. After another lymph node metastasis of the right groin, a lymph node dissection of the right pelvic region and an adjuvant radiotherapy (48Gy) was performed. In August 2018, the patient developed three subcutaneous in-transit metastases on the left lower limb again (**Figure 3A**). Due to the recurring in-transit metastases, we offered a systemic therapy, but the patient refused it. Therefore, we decided to start a treatment with T-VEC injections. The therapy was well tolerated and the metastases partly regressed (**Figure 3B**, **Supplementary Table 1**). Nonetheless, more in-transit metastases developed and one subcutaneous metastasis progressed. We discussed treatment with ICI, but the patient refused intravenous applications. Therefore, we considered an off-label treatment with a MEKi because of the *NRAS* Q61L mutation. The patient consented and trametinib 2mg per day was initiated in combination with T-VEC injections. After four months of the combination therapy, four of five metastases were no longer detectable. One metastasis showed a fibrosed morphology in the ultrasound examinations (**Figure 3C**). The CT scan showed stable lymph nodes. Due to progressive lymphoedema of the limbs after bilateral lymph node dissections and subsequent cardiac decompensation, the treatment with trametinib was paused in July 2019 and T-VEC was stopped in August. After three months, the patient developed new lymph node metastases in the aortal, the left parailiac and the right pelvic region as well as new subcutaneous in-transit metastases. A treatment with nivolumab (qw4) was initiated. After three months, the patient presented with an immune-related colitis and nivolumab was paused. The staging examinations showed a stable disease until August 2020, when a progressive lymph node metastasis with infiltration of the vena cava was found. A radiotherapy of the abdominal lymph node metastases was performed and best supportive care was agreed with the patient who died in May 2021.

## 3 Discussion

We treated three patients with multiple cutaneous and subcutaneous satellite and in-transit metastases and proven *NRAS* mutation with the combination of a MEKi (cobimetinib and trametinib) and intralesional T-VEC injections. The cutaneous and subcutaneous metastases were all regressive under the combination therapy (**Figures 1**–**3**). One patient (case 1) had a full regression of local and locoregional metastases and remained free from progression until date, for almost three years. In this case, the treatment with cobimetinib alone had only led to a regression of locoregional lymph node metastases, with subcutaneous metastases growing further. The combination of cobimetinib and T-VEC led to a durable complete response. This achievement was especially positive because the patient had been suffering from recurring in-transit metastases for over five years and previous treatments with ICI were not able to stop the growth. Unfortunately, in case 2, the patient showed progressive cerebral and locoregional lymph node metastases after three months of combination treatment. Eight weeks after one T-VEC injection, there was no local sign of response to the treatment. After we started the treatment with trametinib and T-VEC, there was a regression of the cutaneous metastases under the combination and a resection of the remaining and disturbing metastases was possible (**Figures 2C, D**). The patient was exempted from painful metastases and regained a better quality of life. Next to the regression of subcutaneous metastases, the third patient (case 3) showed stable distant lymph node metastases for seven months under the combination therapy. Here, the treatment had been started with T-VEC due to recurring in-transit metastases, resulting in a partial response. Due to one progressive metastasis and new in-transit metastases, the treatment was expanded with trametinib and a complete response of subcutaneous metastases could be reached. In conclusion, the combination treatment of T-VEC with trametinib led to a regression of subcutaneous metastases which had been progressive under the treatment with T-VEC alone. All of the patients benefited from the combination treatment with MEKi and intralesional T-VEC. The combination was well tolerated. Two patients reported several episodes of chills and fever after the T-VEC injections which are known common side effects. One patient presented a rash typically seen under treatment with MEKi in combination with BRAF-inhibitors. Hyperkalemia was seen in one of the patients and disappeared in the control examinations. Oedema of the limbs occurred in all of the patients over the course of time, combined with inflammation of the skin and subcutaneous tissue in two patients. The oedema was probably also due to previous lymph node dissections and radiotherapy of the inguinal region in all three patients.

The treatment with T-VEC for unresectable cutaneous, subcutaneous and nodal lesions in patients with melanoma showed a durable response rate of 25% with 17% of patients presenting a complete response in the OPTiM trial (4). Real-world data on T-VEC treatment in stage IIIB – IVM1A melanoma revealed varying complete response rates ranging from 11% to 37% (5). Mostly, cutaneous lesions located on an extremity were treated with T-VEC, similar to our patients. The benefit from T-VEC injections is undisputable but also limited. Therefore, the combination of T-VEC with other treatment options seems an attractive method. Clinical reports have shown several promising outcomes in patients receiving other systemic treatments concurrent with T-VEC, particularly in combination with ICI (5). However, the phase III trial (MASTERKEY-265), evaluating the combination of T-VEC with pembrolizumab, indicated no significant improvement of overall survival (OS) or progressive free survival (PFS) (6). To our knowledge, just one phase IB clinical trial is exploring the combination of T-VEC with BRAF- and MEKi with no results published yet (NCT03088176).

The treatment with MEKi in patients with *NRAS* mutations is not approved by the EMA and FDA and therefore off-label. In particular, the MEKi binimetinib has been tested in a phase II clinical trial. Compared to dacarbazine, binimetinib showed an improved overall response rate of 15% vs. 7% and PFS of 2.8 months vs. 1.5 months. However, there were no significant benefits for the OS (7). Because of these findings, binimetinib was not approved for monotherapy of *NRAS*-mutated melanomas. Thus, in daily clinical routine, patients with *BRAF* wildtype and *NRAS* mutation who had been previously treated and progressed under ICI often have no other option than being treated with a MEKi. Since the demand for new treatment opportunities is high, a number of trials for *NRAS*-mutated melanoma patients are currently focussing on combination therapies (NCT04835805, NCT03973151, NCT03932253, NCT05340621).

To the best of our knowledge, no clinical evidence has been published in the literature about the combination of T-VEC and MEKi as a treatment for metastasized melanoma yet. However, Bommareddy et al. described a synergistic effect of this combination *in vitro* (3). T-VEC together with trametinib led to an increased melanoma cell death *in vitro* due to increased viral replication and apoptosis. Furthermore, they observed reduced tumor growth and enhanced survival in a mouse model treated with trametinib and T-VEC compared to either of these agents alone. This tumor regression was proven to be dependent on activated CD8+ T cells and Batf3+ dendritic cells (3).

## 4 Conclusion

We treated three patients with *NRAS*-mutated metastasized melanoma with the combination of T-VEC and a MEKi. Our case series demonstrates that the combination is a reasonable treatment option which can lead to a long-term anti-tumor response. Two of our three patients showed a complete response of subcutaneous metastases under the combination treatment whereas single treatment with one of the agents only resulted in a partial response. The third patient presented a partial response of rapidly growing cutaneous metastases and experienced a subsequent increased quality of life. The therapy was well tolerated with common adverse events known of each single agent. We suggest the combination of T-VEC and MEKi especially for patients with *NRAS* mutations and uncontrolled in-transit or satellite metastases. Since there is an urgent need for more therapeutic options in *NRAS*-mutated melanoma patients, the combination of T-VEC and MEKi should be investigated in clinical trials to evaluate the efficiency and safety in larger cohorts.

## Data availability statement

The original contributions presented in the study are included in the article/**Supplementary Material**. Further inquiries can be directed to the corresponding author.

## Ethics statement

Ethical review and approval were not required for the study on human participants in accordance with the local legislation and institutional requirements. The patients/participants provided their written informed consent to participate in this study. Written informed consent was obtained from the individual(s) for the publication of any potentially identifiable images or data included in this article.

## Author contributions

SS: assembly of data, data analysis and interpretation, manuscript writing. VM: provision of patients, collection of data, data analysis and interpretation. JU: conception/design, provision of patients, final approval of manuscript. All authors contributed to the article and approved the submitted version.
