# Supplementary material for: Case report: Therapeutic potential of T-VEC in combination with MEK inhibitors in melanoma patients with NRAS mutation
Source: Front Oncol. 2023 Mar 7;13:1111119. doi: 10.3389/fonc.2023.1111119 (PMC10027906; doi:10.3389/fonc.2023.1111119)
Supplement: Supplementary file 1 [file Table_1.docx]

**Supplementary Table 1. Exemplary sizes of metastases and injection volumes during administration of T-VEC**

|  | size of metastasis #1 | injected volume | size of metastasis #2 | injected volume | size of metastasis #3 | injected volume |
| --- | --- | --- | --- | --- | --- | --- |
| **Case 1** | | | | | | |
| 1st injection  **(Fig 1A)** | 11.5 x 11.6 x 7.6 mm | 0.5ml 10^6^ PFU/ml | 4.4 x 5.5 x 2.4 mm | 0.5ml 10^6^ PFU/ml |  |  |
| 2nd injection | 11.4 x 12.3 x 9.6 mm | 0.5ml 10^8^ PFU/ml | 5.8 x 3.5 x 6.1 mm | 0.5ml 10^8^ PFU/ml |  |  |
| 7th injection | 7.7 x 8.4 x 6.9 mm | 0.5ml 10^8^ PFU/ml | 3.9 x 4.2 x 2.2 mm | 0.1ml 10^8^ PFU/ml |  |  |
| 10th injection | 6.4 x 7.9 x 5.7 mm | 0.5ml 10^8^ PFU/ml | not detectable anymore |  |  |  |
| 20th injection  **(Fig 1B)** | 4.3 x 5.6 x 4.2 mm | 0.5ml 10^8^ PFU/ml |  |  |  |  |
| 34th injection  **(Fig 1C)** | 3.8 x 5.3 x 3.4 mm | 0.5ml 10^8^ PFU/ml |  |  |  |  |
| **Case 2** | | | | | | |
| 1st injection  **(Fig 2B)** | 21mm | 1ml 10^6^ PFU/ml | 18mm | 1ml 10^6^ PFU/ml | 25mm | 1ml 10^6^ PFU/ml |
| 3rd injection  **(Fig 2C)** | 23mm | 1ml 10^8^ PFU/ml | 15mm | 1ml 10^8^ PFU/ml | 23mm | 1ml 10^8^ PFU/ml |
| 10th injection | 22mm | 1ml 10^8^ PFU/ml | 13mm | 1ml 10^8^ PFU/ml | 18mm | 1ml 10^8^ PFU/ml |
| **Case 3** | | | | | | |
| 1st injection  **(Fig 3A)** | 6.6 x 8.6 x 10.9 mm | 0.5ml 10^6^ PFU/ml | 3.8 x 2.9 x 3.9 mm | 0.1ml 10^6^ PFU/ml |  |  |
| 3rd injection | 14.6 x 13.9 x 10.9 mm | 0.5ml 10^8^ PFU/ml | 6.0 x 7.9 x 3.2 mm | 0.5ml 10^8^ PFU/ml |  |  |
| 12th injection  **(Fig 3B)** | 19.1 x 16.9 x 12.9 mm | 1ml 10^8^ PFU/ml | 3.3 x 3.1 x 2.1 mm | 0.1ml 10^8^ PFU/ml |  |  |
| 14th injection | 18.7 x 16.0 x 12.2 mm | 1ml 10^8^ PFU/ml | not detectable anymore |  |  |  |
| 20th injection **(Fig 3D)** | 18.2 x 15.8 x 12.4 mm | 1ml 10^8^ PFU/ml |  |  |  |  |

Abbreviation: PFU, plaque-forming units
